# The Effect of Social Support on Depression among Economically Disadvantaged College Students: The Mediating Role of Psychological Resilience and the Moderating Role of Geography

**DOI:** 10.3390/ijerph20043053

**Published:** 2023-02-09

**Authors:** Xianglian Yu, Fen Xiong, Hanbing Zhang, Zhihong Ren, Lianzhong Liu, Lin Zhang, Zongkui Zhou

**Affiliations:** 1 Department of Psychology, Central China Normal University, Wuhan 430079, China; 2 Department of Education, Jianghan University, Wuhan 430056, China; 3 Key Laboratory of Adolescent Cyberpsychology and Behavior, Ministry of Education, Wuhan 430079, China; 4 Key Laboratory of Human Development and Mental Health of Hubei Province, Wuhan 430079, China; 5 School of Psychology, Fujian Normal University, Fuzhou 350108, China; 6 Wuhan Mental Health Center, Wuhan 430022, China

**Keywords:** social support, psychological resilience, depression, economically disadvantaged college students, moderated mediation

## Abstract

The study examined the influence of social support on depression, including the mediating role of psychological resilience and the moderating role of geography. Questionnaires were completed by 424 economically disadvantaged college students in two provinces, X, a coastal province, and Y, an inland province. The results indicated that (1) the social support of economically disadvantaged college students was positively correlated to psychological resilience (*β* = 0.62, *t* = 11.22, *p* < 0.001); (2) the psychological resilience of economically disadvantaged college students was negatively correlated with depression (*β* = −0.24, *t* = −10.3, *p* < 0.001); (3) the social support of economically disadvantaged college students was negatively correlated with depression (*β* = −0.08, *t* = −2.85, *p* < 0.001); (4) the psychological resilience of economically disadvantaged college students played a partial mediating role between social support and depression; and (5) geography played a moderating role in the effect of social support on depression.

## 1. Introduction

On 25 February 2021, China achieved a comprehensive victory in the battle against poverty, making a crucial contribution to the task of achieving the goal of building a moderately prosperous society [1]. However, the solution to poverty is not only to solve financial problems but also to solve a series of psychological problems caused by material poverty. Specifically, it is necessary to help the low-income population build confidence and make them fully aware of the importance of motivation. Coupled with the reform of China’s education system in recent years, many adults who come from low-income families enter university. The proportion of these people has been increasing year after year. However, the mental health problems of economically disadvantaged college students are not effectively addressed in the short term. Through a survey of economically disadvantaged college students in old revolutionary areas, a study found that, due to family economic situations, economically disadvantaged college students were unable to meet their most basic survival needs, felt inferior, and became lonely, apathetic, and anxious [2].

Physiologically, college students generally belong to the late adolescent period, which is the transition stage from adolescence to adulthood. During this period, physical, cognitive, and social aspects change greatly. These changes can unbalance physical and mental development, which can influence various contradictions in psychological development. If the contradictions are not handled properly or if there are not enough psychological resources to resolve the contradictions, affected people can become doubtful, confused, experience negation, and fall into a negative psychological state. Many factors have an impact on depression in college students, including genes, personality, family, school, and social factors. For example, in facing difficulties and stress, social support can improve college students’ confidence and courage, thus reducing depression [3]. A low psychological resilience makes college students less flexible and negative in coping with stressful life events, and affected students are more likely to become depressed [4]. A meta-analysis found good family economic status was one of the important protective factors against depression [5].

### 1.1. Social Support and Depression

Depression is an important indicator of people’s mental health. As a negative emotion, depression has serious impacts on people’s physical and mental health. Depressed people not only have various symptoms such as a depressed mood, slowed thinking, and decreased appetite, but may even engage in behaviors such as self-harm and suicide [6]. After entering university, economically disadvantaged college students face many students from different places. With different backgrounds and family conditions, these students may experience greater psychological pressure. They may take measures of avoidance or marginalization in interpersonal communication [7], and inevitably have higher levels of depression.

Social support refers to the resources that people receive from social activities, including material or emotional help from family and friends [8]. It is generally believed that social support can be divided into two categories: objective support and subjective support. Objective support includes material assistance and direct services. Subjective support refers to the emotional experience of feeling respected, understood, and supported by others [9]. Social support theory suggests that the stronger the social support network that people have, the better they can handle problems. For example, emotional and material support from friends and family can improve people’s ability to cope with stressful events, and in turn, lower levels of depression will be experienced. Many studies found that if people had high social support scores, their depression scores would be low [10]. Compared to people with high household incomes, low-income groups receive less social support [11].

### 1.2. The Mediating Role of Psychological Resilience

The definition of psychological resilience has since not reached a full consensus. There are three main opinions. Some researchers consider psychological resilience as a result of maintaining function after experiencing stress, while other researchers suggest that psychological resilience is the ability of individuals to maintain a relatively stable, healthy level of psychological and physical functioning when exposed to isolating and potentially highly disruptive events [12]. Alternatively, a third view associates psychological resilience with a process by which people adapt well to facing life adversity, trauma, tragedy, threat, and other major life stressors and recover from difficult experiences, which is the definition used in this study [13].

Psychological resilience can positively correlate with social support. Through a survey of healthcare workers in Wuhan, a study found that social support had a high positive correlation with psychological resilience [14]. In addition, a study on migrant older adults showed a significant association between social support and psychological resilience [15].

Psychological resilience can also negatively correlate with depression. Researchers believe constructing high psychological resilience was beneficial in reducing depression in adolescents [16]. A previous study investigated the psychological status of Spanish university students and found that people with high psychological resilience scores showed fewer depressive symptoms [17]. By analyzing the relationship between jealousy, depression, social support, and psychological resilience, one study found that psychological resilience was not only negatively correlated with jealousy but was also negatively correlated with depression [3].

The above study shows a correlation between social support, psychological resilience, and depression. Psychological resilience can be a mediator between environmental or individual factors and emotions, which has been examined by many previous studies. For example, one previous study found that psychological resilience in adolescent females partially mediated the relationship between social support and negative affect [16]. Another previous study found that psychological resilience played a mediating role in the effect of empathic competence on depression in clinical medical students [18].

### 1.3. The Moderating Role of Geography

Geography can moderate the association between social support and depression. Firstly, according to psychologist Urie Bronfenbrenner, the mental health of an individual is influenced by the interaction of four levels of environmental factors: microsystem, mesosystem, exosystem, and macrosystem. Geography, which belongs to the macrosystem, and social support, which belongs to the microsystem can interact with each other and influence the mental health of an individual [19]. Secondly, the disparities in the level of economic development by geography can moderate the relationship between social support and depression. A previous study found that college students from wealthy families were associated with a lower risk of depression compared with their counterparts from low-income families [20]. Another study found that the well-being of residents in the high-income region was higher than that in low-income regions [21]. Subjective well-being, as an important psychological indicator of mental health, has been shown to correlate with depression [22].

### 1.4. Hypothesis

**Figure 1:**
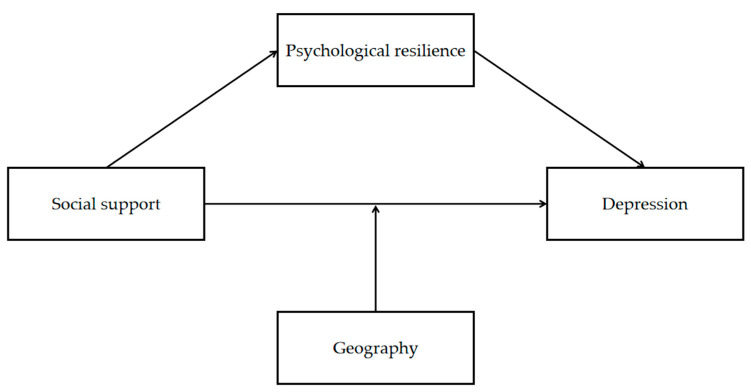
The constructed moderated mediating model.

As a special group in colleges, economically disadvantaged college students are under great psychological and economic pressure, but they lack the attention of researchers. Therefore, it is of urgent practical significance to explore the mechanism of economically disadvantaged college students to deal with psychological problems. Thus, this study proposes the following hypotheses (as shown in Figure 1):

**H1.**  *Social support* *for* *economically disadvantaged college students is negatively associated with depression.*

**H2.**  *The psychological resilience of economically disadvantaged college students negatively correlates with depression.*

**H3.**  *Psychological resilience plays a mediating role between the social support and depression of economically disadvantaged college students.*

**H4.**  *Geography plays a moderating role between the social support and depression of economically disadvantaged college students.*

## 2. Materials and Methods

### 2.1. Subjects

In this study, the method of cluster random sampling was adopted. With one online platform as the medium, we contacted college teachers and students through WeChat, QQ, and other network platforms. Four hundred thirty-three economically disadvantaged college students from a coastal city and a city in Central China were recruited to complete questionnaires. In terms of economic development level, the coastal city was more developed than the city in Central China. In terms of the impact of the COVID-19 pandemic, the city in Central China, the place where the pandemic first broke out, has a higher impact severity than the coastal city. After a quality audit, 424 valid questionnaires were included in the analysis, with an effective rate of 98%.

### 2.2. Materials

#### 2.2.1. Social Support

Perceived social support scale (PSSS) was used to access social support in the study, including family support (e.g., “my family tries to help me”), friend support (e.g., “my friends try to help me”), and other support [23]. The questions were scored using 12 items on a 7-point Likert scale, with 1 indicating “strongly disagree” and 7 meaning “strongly agree”. The higher the total score of the items, the higher the perceived social support. The Cronbach’s alpha was 0.95.

#### 2.2.2. Depression

Depression was accessed by the self-rating depression scale (SDS). According to symptoms frequency, the questions were scored using 20 items on a 4-point Likert scale, with 1 indicating “rarely or not at all” and 4 indicating “persistent” (e.g., “I feel downhearted and blue”) [24]. Among these items, 10 items were positive scores and 10 items were reverse scores. The higher the total item score, the more severe the depressive state the subject had. The Cronbach’s alpha was 0.83.

#### 2.2.3. Psychological Resilience

The study used the Connor–Davidson Resilience Scale (CD-RISC). The questions were scored using 25 items on a 5-point Likert scale, with 1 meaning “never” and 5 meaning always (e.g., “coping with stress makes me stronger”) [25]. The CD-RICS includes five factors and describes different qualities such as strengthening effects of stress, trust, tolerance of negative affect, hardiness, self-efficacy, and so on [26]. The scale is used to measure the psychological resilience of the subject. The higher the total score of the items, the more psychological resilience the subject has. The Cronbach’s alpha for this scale in this study was 0.96.

### 2.3. Data Analysis

In this study, data were analyzed using SPSS version 26.0 software and the SPSS macro program PROCESS version 3.3. Firstly, in this study, because the data were self-reported, Harman’s single-factor test was used to test common method bias. Secondly, descriptive statistics and correlation analysis were conducted for social support, depression level, and mental resilience. Finally, PROCESS in SPSS26.0 was used to test the hypotheses. Model 4 was used to test the proposed mediating effect, adopting 5000 bootstrapping samples. We used model 5 to test the moderated mediating effect, considering social support as an independent variable and depression as a dependent variable.

## 3. Results

### 3.1. Common Method Biases

Harman’s single-factor test was used and the results showed that there were a total of eight factors with eigenvalues greater than 1 and the first principal factor explained 34.90% of the variance, which was less than the 40% threshold. Therefore, the common method bias in this study was not serious. This indicated that there was no serious common method bias among the variables.

### 3.2. Descriptive Analysis and Correlation Analyses

Table 1 shows the results of descriptive statistics. There were 424 subjects in this study, and 205 subjects were from Province X, while 219 subjects were from Province Y, including 301 women and 123 men. Among them, 74 participants were freshmen, 78 participants were sophomores, 161 participants were juniors, and 111 participants were seniors. In addition, 138 participants had been class officers while 286 had not been class offices.

The results of correlations showed that social support was positively correlated with psychological resilience (r = 0.497, *p* < 0.01). Psychological resilience (r = −0.538, *p* < 0.01) and social support (r = −0.342, *p* < 0.01) were negatively correlated with depression. In addition, geography was positively correlated with depression (r = 0.105, *p* < 0.05) (Table 2).

### 3.3. Test for Mediating Effects

Model 4 in PROCESS was used to test the mediating effects of psychological resilience. The results showed that social support significantly positively correlated with psychological resilience (*β* = 0.62, *t* = 11.22, *p* < 0.001), psychological resilience negatively correlated with depression (*β* = −0.24, *t* = −10.18, *p* < 0.001), and social support negatively correlated with depression (*β* = −0.23, *t* = −7.78, *p* < 0.001) (Table 3). The direct effect of social support on depression remained significant after the inclusion of the mediating variable of psychological resilience. Thus, psychological resilience partially mediated the effect of social support on depression (indirect effect = −0.146, BootSE = 0.02, BootCI = (−0.188, −0.109)).

### 3.4. Test for Moderated Mediation

This study used Model 5 in PROCESS to test the moderated mediation effect. The results showed that the interaction term between social support and geography was positively related to depression (*β* = 0.11, t = 2.1, *p* < 0.05). This showed that geography moderated the effect of social support on depression (Table 4).

**Figure 2:**
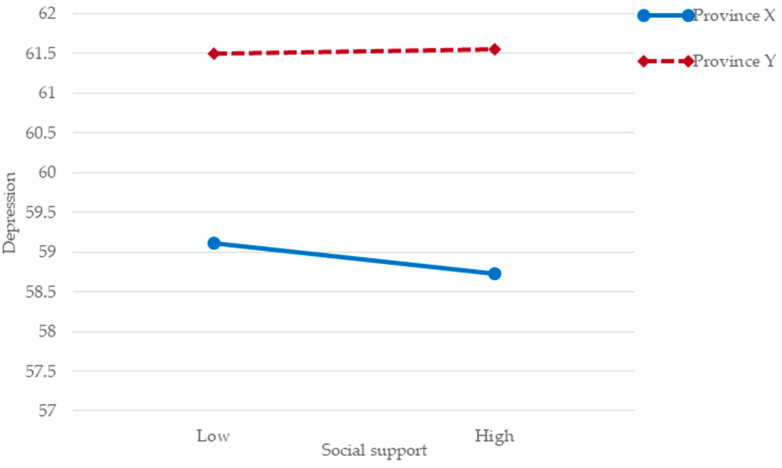
The moderating effect of geography among economically disadvantaged college students from provinces X and Y.

To further analyze the moderating effect of geography, a simple slope test was used to examine the moderating effect of geography on social support and depression. The results showed (Figure 2) that social support negatively correlated with depression when subjects were from a coastal province X (*t* = −3.41, *p* = 0.007 < 0.01), and social support did not significantly correlate with depression among economically disadvantaged college students when subjects were from the inland province Y (*t* = −0.84, *p* = 0.4 > 0.05).

## 4. Discussion

### 4.1. The Relationship between Social Support and Depression among Economically Disadvantaged College Students

This study found that social support for economically disadvantaged college students negatively correlated with personality formation. In this period, self-concept and self-esteem were further refined, and self-differentiation to self-integration was experienced [27]. However, economically disadvantaged college students were embarrassed by economic conditions and had relatively low social status. When these students entered university, they may develop psychological problems, such as compulsion, depression, interpersonal sensitivity, hostility, and life distress, which may also worsen their psychological health compared to non-disadvantaged students [28]. Social support is an important protective factor for psychological health. It is generally believed that there are two mechanisms for the effect of social support on physical and mental health: the main effect model and the buffering effects model [29]. The former suggests that high social support also means a high level of mental health, while the buffering model suggests that social support is similar to a buffer that buffers the negative effects of various negative events on physical and mental health. Combined with the negative correlation between social support on depression in the present study, we could understand that high social support implies high levels of mental health [30]. Social support reduces the likelihood of depression by buffering the negative effects and maintaining a good emotional experience [31].

### 4.2. The Mediating Role of Psychological Resilience

In addition, this study found a partially mediating effect of psychological resilience between social support and depression. Social support is negatively correlated with psychological resilience [32] and psychological resilience is negatively correlated with depression [33]. The effect of social support on depression can work through a direct pathway or the mediating role of psychological resilience. Psychological resilience plays a mediating role between social support and depression [34]. As an important protective factor against psychological problems, psychological resilience not only directly affects the psychological health of economically disadvantaged college students but also regulates mental health problems [35]. Specifically, on the one hand, social support allows people to cope with negative events and negative emotions through the resources they receive from social activities, including material or mental help from family, and friends. On the other hand, social support, through creating a caring, considerate, understanding, and accepting atmosphere, can implicitly help college students develop some psychological resilience traits, which help them increase self-confidence and the courage to cope with negative events and negative emotions [29].

The results of this study support the mental resilience framework. This framework holds that the effect of psychological resilience is manifested through a series of interaction processes, including the interaction between risk factors and protective factors in the environment, between people and the environment, and between the situation and internal psychological resilience. The last process may lead to three kinds of results: resilience reorganization involves becoming stronger and reaching a higher level of resilience. The reorganization of homeostasis involves a return to an initial state that existed long before the stress or danger occurred. Maladaptive recombination indicates a failure to show mental resilience, that is, the mental function remains at a very low level [36].

Specifically, in this study, material distress perceived by economically disadvantaged college students was a risk factor, while social support was a protective factor. In the first process, perceived economic hardship interacted with social support, which served as a buffer and an important external protective factor. When people interacted with the environment, if the social support was strong enough, selective awareness may form to create a relatively protective environment. The protective environment created would increase the possibility of restructuring psychological resilience in the third process. It is helpful to maintain mental function at a high level, alleviate the impact of economic poverty on students with economic difficulties, and reduce levels of depression.

### 4.3. The Moderating Role of Geography

This study also found that the geography of economically disadvantaged college students moderated the effect of social support on depression. The results of the moderating effect test showed that social support was a significant negative predictor of depression for subjects from coastal province X. For subjects from an inland province Y, social support was not a significant negative predictor of depression for economically disadvantaged college students. Social support for economically disadvantaged college students from an inland province Y did not work well to mitigate and prevent psychological disorders caused by stressful life events and protect psychological health [37,38].

This discrepancy can be understood in the context of COVID-19. The outbreak of COVID-19 posed a serious threat to the lives of people in province Y. On 30 July 2020, the cumulative data of confirmed COVID-19 patients in province Y was controlled and released as 68,135 cases and 4512 deaths, which was the hardest hit area of COVID-19 in China. Province X reported a cumulative total of 296 confirmed local cases and one death. During the outbreak, with various rumors and stigmatization on the Internet [39], province Y was subjected to a greater impact of online public opinion, and the detection rate of depression and anxiety among college students in the capital city of province Y was higher than that in other regions [40]. People of province Y were also prone to numbness after two months of isolation, causing disorders in their psychological functions. Maslow’s hierarchy of needs theory states that the human organism is a security-seeking mechanism, and if people’s security needs are severely disrupted, they cannot maintain psychological balance. When people realize that they are incapable of coping with the excessive demands of the situation, people are highly susceptible to a pessimistic state of mind, which can further reduce the efficiency of one’s cognitive activities and cause one to lose confidence and hope.

This study verifies the buffering effect model of social support. The theory suggests that social support has a beneficial buffer effect on the stress suffered by people under stress. In this model, social support buffers the impact of stressful events on health and protects people from the damage of stress [41]. In response to the severe epidemic results, measures were taken to help people to have greater access to social support, material help, behavioral assistance, intimate interaction behaviors, and positive social interactions. Through an empirical study, researchers found that people had a higher level of social support before the epidemic than after. The buffering effect of social support was dysfunctional, and people were more likely to make a false assessment of potentially stressful events due to their negative state of mind [7].

## 5. Limitations and Practical Implications

Despite the above conclusions of the study and the richness of research on the mental health of economically disadvantaged college students, there are still some shortcomings in this study. First, the sample of this study contains more female subjects than male subjects. Although the female population occupies a larger proportion of the low-income population, we still cannot conclude that larger proportions of economically disadvantaged college students in provinces X and Y are female, which may have some influence on the results of this study. Second, the data were obtained from subjects’ self-reports, which may have impacts on some bias considering the influence of the social approval effect and the satisfaction principle of decision making [42]. Third, the results of this study are only applicable to economically disadvantaged college students. Further research is needed to determine whether psychological resilience mediates the effect of social support on depression in other groups and whether geography moderates the effect of social support on depression. Finally, the study is exploratory and the study design is cross-sectional. Therefore, authors need to take care not to go further than the “potential” role of the proposed relationships.

Over the past hundred years, psychologists have focused on psychological problems, mental illness and its treatment, and other pathologic issues, but paid little attention to the pursuit of happiness for normal people. With the rise of positive psychology, more and more psychologists began to pay attention to the positive qualities of people. They hoped to shift the focus from the worst things in life to the best things in life and used scientific methods to explore the positive psychological factors for the prosperity of people, groups, and communities [43]. This is especially true for economically disadvantaged college students. Although some studies have examined the relationship between social support, psychological resilience, and depression, most of these studies focused on general college students and secondary school students. These studies have not revealed the mechanism of the role of psychological resilience among economically disadvantaged college students between social support and depression. Their economic distress is related to the psychological burden and negative self-schema, which make them bear more negative emotions. Therefore, for affected individuals, it is necessary to cultivate positive cognition, positive emotional experience, positive organizational structure, and other positive psychological resilience, which can help them form a strong buffer zone for negative events and reduce the impact of negative events.

## 6. Conclusions

The findings demonstrated that social support for economically disadvantaged college students negatively correlated with depression, and the psychological resilience of economically disadvantaged college students was negatively correlated with depression. Additionally, psychological resilience played a partially mediating role in the effect of social support on depression held by economically disadvantaged college students, and geography played a moderating role in the effect of social support on depression.

## Figures and Tables

**Table 1:** Descriptive statistics of the participants (N = 424).

Variables	Levels	Frequency	Percentage
Gender	Male	123	29.01%
	Female	301	70.99%
Grades	Freshman year	74	17.45%
	Sophomore year	78	18.40%
	Junior year	161	37.97%
	Senior year	111	26.18%
Majors	Literature and history	138	32.55%
	Science	77	18.16%
	Engineering	72	16.98%
	Art	45	10.61%
	Sports	17	4.01%
	Other	75	17.69%
Being class officer	Yes	138	32.55%
	No	286	67.45%
Geography	Province X	205	48.35%
	Province Y	219	51.65%

**Table 2:** Mean, standard deviations, and correlations among variables (N = 424).

	M	SD	1	2	3
1. Social support	65.802	12.811			
2. Psychological resilience	85.540	16.469	0.497 **		
3. Depression	39.495	8.135	−0.342 **	−0.538 **	
4. Geography	0.517	0.500	−0.021	−0.038	0.105 *

* *p* < 0.05, ** *p* < 0.01.

**Table 3:** The mediating effect of psychological resilience (N = 424).

	Depression			Psychological Resilience			Depression		
	β	SE	t	β	SE	t	β	SE	t
Social support	−0.23	0.03	−7.78 ***	0.62	0.05	11.27 ***	−0.08	0.03	−2.70 **
Gender	0.61	0.82	0.74	−2.93	1.54	−1.90	−0.08	0.74	−0.11
Being class officer	0.33	0.79	0.42	−3.02	1.49	−2.02 *	−0.38	0.71	−0.54
Psychological resilience							−0.24	0.02	−10.18 ***
R^2^	0.13			0.24			0.3		
F	20.42 ***			45.00 ***			44.95 ***		

*** *p* < 0.001, ** *p* < 0.01, * *p* < 0.05.

**Table 4:** A test of the moderated mediation effect (N = 424).

Predictors	Depression				Psychological Resilience				Depression			
	β	SE	t	95% CI	β	SE	t	95% CI	β	SE	t	95% CI
Social support	−0.23	0.03	−7.78 ***	[−0.28, −0.17]	0.62	0.05	11.27 ***	[0.51, 0.73]	−0.08	0.03	−2.85 ***	[−0.14, −0.03]
Gender	0.61	0.82	0.74	[−1.00, 2.22]	−2.93	1.54	−1.90	[−5.97, 0.1]	0.26	0.75	0.35	[−1.20, 1.73]
Class officer	0.33	0.79	0.42	[−1.23, 1.89]	−3.02	1.49	−2.02 *	[−5.95, −0.08]	−0.49	0.71	−0.69	[−1.88, 0.91]
Psychological resilience									−0.24	0.02	−10.30 ***	[−0.28, −0.19]
Geography									1.30	0.67	1.94	[−0.02, 2.63]
Social support×Geography									0.11	0.05	2.10 *	[0.01, 0.21]
R^2^	0.13				0.24				0.31			
F	20.42 ***				45.00 **				31.76 ***			

*** *p* < 0.001, ** *p* < 0.01, * *p* < 0.05.

## Data Availability

The raw data of the present study are available from the corresponding author upon reasonable request.
